# A Cross-Sectional, Descriptive Qualitative Study of Information Counselling During Tuberculosis Treatment in South Africa: Awareness of XDR-TB Patients on Ototoxic Effects

**DOI:** 10.3390/ijerph22010091

**Published:** 2025-01-10

**Authors:** Skyla Maria Arendse, Katijah Khoza-Shangase

**Affiliations:** School of Human & Community Development, Faculty of Humanities, University of the Witwatersrand, Johannesburg 2050, South Africa; skylaarendse123@gmail.com

**Keywords:** ototoxicity, extensively drug-resistant tuberculosis, information counselling, audiological monitoring, South Africa, patient awareness, side effects management, tele-audiology, healthcare pathways, tuberculosis treatment

## Abstract

Ototoxicity is a significant adverse effect associated with second-line anti-tuberculosis (TB) medications, particularly in treating extensively drug-resistant TB (XDR-TB). This study investigated the awareness of ototoxic effects among adults with XDR-TB undergoing treatment in South Africa, specifically exploring the role of information counselling on ototoxic symptoms, the timing of counselling, the content covered, and the management pathways available. This cross-sectional, descriptive qualitative study was conducted at Brooklyn Chest Hospital in the Western Cape. Ten adults with XDR-TB were purposively sampled and participated in semi-structured in-depth interviews. Data were thematically analyzed and the results revealed variability in information counselling on ototoxicity, with only 30% of participants receiving comprehensive counselling that specifically addressed ototoxic symptoms. The timing of counselling was inconsistent: while 70% of participants received some information before treatment, the remainder received counselling only after treatment initiation, which may have impacted early symptom recognition. Participants’ awareness of ototoxic symptoms was generally limited, with most identifying hearing loss but few recognizing other symptoms such as tinnitus or dizziness. Furthermore, only 20% of participants were provided with clear referral pathways for symptom management. These findings highlight a gap in the depth, timing, and specificity of information counselling on ototoxic effects for XDR-TB in this context. Several interventions can be implemented to address this gap.

## 1. Introduction

Tuberculosis (TB), caused by *Mycobacterium tuberculosis*, is a leading cause of morbidity and mortality worldwide and has become especially prevalent in South Africa [1,2]. Ranked among the top eight countries with the highest TB burden, South Africa faces a dual epidemic of TB and HIV, deepening the challenges of managing the disease [2]. Compounding these challenges is the emergence of drug-resistant strains of TB, particularly multidrug-resistant (MDR) and extensively drug-resistant (XDR) TB, which require specialized treatment approaches [3]. XDR-TB, which shows resistance to both first-line drugs and at least one Group A drug such as fluoroquinolones, necessitates prolonged and complex treatment regimens, often with second-line anti-TB drugs known for their ototoxic properties [3]. These ototoxic medications, while lifesaving, can lead to significant auditory and vestibular side effects, further complicating patient care. This risk is especially relevant in South Africa, where many public healthcare facilities still rely on aminoglycosides, injectable antibiotics to treat drug-resistant TB [4].

Ototoxicity, defined as drug-induced damage to the auditory or vestibular system, is a significant concern for XDR-TB patients undergoing treatment with aminoglycosides or other ototoxic drugs [5,6]. This adverse effect arises from cellular degeneration in the cochlear and vestibular structures, which can lead to permanent hearing loss, balance dysfunction, or both [7]. Studies have shown that aminoglycosides can destroy cochlear hair cells, leading to high-frequency hearing loss that can progressively impact speech frequencies [8]. This progression can severely affect patients’ ability to communicate, work, and engage in social interactions, further complicating their health and quality of life. Without timely detection and management, these side effects can affect their mobility and overall quality of life —over and above severely impairing patients’ communication—hindering their ability to adhere to lengthy treatment regimens. Ototoxicity in TB treatment is particularly problematic because it often goes unnoticed until significant damage occurs, largely due to the initial high-frequency nature of the hearing loss, which can escape early detection unless proper audiological monitoring is in place [9,10].

In South Africa, the high prevalence of XDR-TB and the continued reliance on aminoglycosides highlights the critical need for patient awareness of ototoxic risks and for proactive audiological management [10]. Central to this management is effective information counselling, which prepares patients to recognize early signs of ototoxicity and seek timely interventions. However, despite recommendations from the Health Professions Council of South Africa (HPCSA) [11] which emphasize the necessity of pre-treatment counselling on the ototoxic effects of TB medications, many patients do not receive adequate information regarding these potential side effects [8,10,12]. This gap in patient education highlights the need for research into current counselling practices and their impact on patient awareness and outcomes. Counselling on ototoxicity involves informing patients of the risk of auditory and vestibular damage, the symptoms to monitor (such as tinnitus, dizziness, and hearing loss), and the importance of reporting these symptoms early. Effective information counselling, provided before and during the treatment process, is crucial not only for preparing patients for potential side effects but also for encouraging adherence to the prescribed TB regimen despite these risks [13,14]. This study investigates these gaps by exploring the extent of patient awareness, the content and timing of counselling, and the barriers to effective communication in a South African context.

A critical issue in the South African context is the lack of standardized, comprehensive information counselling protocols for XDR-TB patients, especially regarding ototoxicity [8,14]. Evidence suggests that counselling practices are inconsistent across healthcare facilities, with many patients receiving general information on TB treatment but lacking specific details on the potential for hearing-related side effects [10,14,15]. Studies have highlighted that a significant number of TB patients remain unaware of the full range of ototoxic side effects of their medications and the impact these can have on their lives [5,10,14,15]. As a result, symptoms like tinnitus and dizziness—often the first signs of ototoxicity—are frequently unreported, which delays diagnosis and intervention. Given that these symptoms may precede irreversible hearing loss, early detection and prompt action are essential to limit the extent of auditory damage and ensure that affected patients can access appropriate interventions.

In addition to inconsistent information counselling, the timing of audiological evaluations for XDR-TB patients in South Africa often falls short of HPCSA guidelines [10,16]. These guidelines recommend a baseline audiological assessment prior to the initiation of ototoxic treatment, followed by regular monitoring throughout treatment. However, in practice, baseline assessments are frequently conducted after treatment has begun, and follow-up testing is often sporadic or absent. This gap can result in missed opportunities to detect and address ototoxic effects early, which would enable healthcare providers to consider alternative treatments or initiate protective measures, such as dose adjustments. Furthermore, in many public healthcare settings, resource constraints and high patient volumes limit the capacity of healthcare providers to deliver comprehensive information counselling or implement regular audiological monitoring [8,10,17]. This situation places XDR-TB patients at an increased risk of untreated ototoxicity, potentially leading to permanent hearing loss and its associated social, emotional, and economic consequences [18].

The provision of information counselling is especially crucial for empowering patients in the South African healthcare context, where factors such as limited healthcare access, language diversity, and variable health literacy levels create additional barriers to effective patient communication [19]. By educating patients on the risks associated with ototoxic medications, healthcare providers can foster a greater sense of autonomy among patients, enabling them to actively participate in their care, recognize early signs of ototoxicity, and seek timely intervention. Comprehensive information counselling also has the potential to improve patients’ adherence to treatment. When patients understand both the benefits and risks of their medications, they are more likely to comply with the full treatment regimen, reducing the likelihood of non-adherence and further drug resistance [19]. This is particularly relevant in cases where the severity of XDR-TB necessitates long-term use of ototoxic medications, and any interruption in treatment could compromise outcomes and exacerbate resistance.

Despite the recognized importance of counselling, studies show that healthcare providers often face limitations in delivering comprehensive information on ototoxicity to TB patients [15,20]. Factors such as high caseloads, time constraints, and limited availability of audiologists contribute to this shortfall [21,22]. Research conducted in South Africa has shown that only a small percentage of healthcare providers regularly counsel their patients on ototoxic risks, often citing workload pressures and resource limitations as barriers [5,8,10,12]. The impact of this shortfall on patients is substantial: inadequate counselling can lead to misunderstandings about treatment, underreporting of symptoms, and delays in accessing necessary support. For XDR-TB patients, who are already coping with a challenging diagnosis and the demands of a rigorous treatment regimen, this lack of information can add to the psychological and emotional strain of their experience.

While previous studies within the South African context have highlighted the prevalence of ototoxic effects in TB treatment [18,23,24,25,26,27,28,29], significant gaps remain in understanding the variability and systemic limitations of patient counselling in South Africa. For instance, evidence suggests that fewer than 40% of patients receive comprehensive counselling on ototoxicity risks before treatment initiation, with disparities in the depth, timing, and cultural relevance of the information provided [8,10,26,27,29,30]. Furthermore, there is limited integration of audiological monitoring into routine care for XDR-TB patients, compounded by linguistic and systemic barriers in public healthcare settings. This study addresses these gaps by exploring patient awareness of ototoxicity and the factors influencing counselling practices, focusing specifically on the South African context. By examining linguistic and cultural barriers, as well as systemic issues, this research provides new insights and actionable recommendations for improving counselling practices and patient outcomes in resource-constrained settings.

The findings from this study have implications beyond the South African context, as ototoxicity is a significant concern in TB treatment worldwide, particularly in high-burden, resource-limited settings, such as India, Ethiopia, Bangladesh, Nigeria, and Pakistan. Globally, aminoglycosides remain a critical component of drug-resistant TB regimens, despite their ototoxic potential. In similar settings, studies have reported variability in the implementation of pre-treatment counselling, monitoring, and patient education, highlighting systemic challenges that compromise adherence to WHO-recommended protocols. For instance, evidence from these countries demonstrates that high numbers of patients on aminoglycosides experience hearing loss, yet structured audiological monitoring remains sparse. By addressing the gaps in counselling and monitoring, this study provides insights that can inform patient-centered TB care and align local practices with global standards to mitigate treatment-related adverse effects.

Given the pressing need to address these gaps in patient education, this study investigates the awareness of adults with XDR-TB in South Africa regarding the ototoxic effects of their TB medications, particularly as gained through information counselling during treatment. This investigation addressed the following specific objectives: (1) to determine if XDR-TB patients received information counselling regarding the ototoxic effects of TB medications; (2) to establish the timing of the information counselling provided to XDR-TB patients; (3) to explore the content of the information counselling provided regarding ototoxic side effects; (4) to describe reported occurrences of ototoxic effects among XDR-TB patients; and (5) to establish the timing of ototoxicity management and follow-up actions, if any, after symptoms were reported. The findings are intended to inform improvements in counselling protocols, with the goal of enhancing patient awareness, fostering early symptom detection, and promoting optimal treatment adherence among XDR-TB patients in South Africa.

## 2. Materials and Methods

### 2.1. Study Design

This study used a cross-sectional, descriptive qualitative design to examine the awareness of ototoxic effects among adults with XDR-TB undergoing treatment in South Africa, focusing specifically on the role of information counselling [31,32]. A qualitative approach was chosen to gain an in-depth insight into participants’ personal experiences, perceptions, and understanding of the information provided on ototoxicity, as well as the timing, content, and management pathways associated with this information. The choice of a cross-sectional, descriptive qualitative design is well-suited for exploring complex, context-dependent phenomena [31,32], such as how XDR-TB patients interpret and engage with information about ototoxicity in a high-burden, resource-limited setting like South Africa. This approach allows for a detailed exploration of patient perspectives that may be influenced by multiple factors, including socio-economic constraints, healthcare accessibility, and cultural or linguistic diversity. This design also enables an in-depth exploration of personal experiences, which are critical for identifying gaps in counselling practices and understanding the barriers to effective communication in a resource-constrained healthcare setting like South Africa. Qualitative research methods are beneficial for understanding how patients perceive the risks associated with ototoxic medications and their reactions to these potential side effects.

The cross-sectional nature of the design meant that data collection was limited to a single point in time, providing a snapshot of the participants’ awareness and experiences at a specific stage of their treatment journey, providing valuable insights into current practices and their potential impacts. This approach aligns with the study’s aim of generating evidence that can inform patient-centered interventions and improve the quality of TB care in similar contexts. Given that XDR-TB treatment is a prolonged process often involving extended hospital stays, this design enabled the study to capture insights from patients with diverse lengths of treatment experience, providing a representative range of counselling and symptom awareness experiences.

### 2.2. Study Setting

The study was conducted at Brooklyn Chest Hospital, a specialized TB treatment facility located in the southwestern sub-structure of the Metro District Health Services in the Western Cape, South Africa. This facility provides both inpatient and outpatient services for drug-sensitive, MDR, and XDR-TB patients. The hospital was selected due to its referral status for complex TB cases, providing a relevant setting for accessing XDR-TB patients who receive ototoxic medications.

### 2.3. Participants

#### 2.3.1. Inclusion Criteria

- Adults aged 18 years or older who were diagnosed with XDR-TB.
- Individuals who were actively undergoing XDR-TB treatment, receiving ototoxic second-line injectable drugs at Brooklyn Chest Hospital, and were resistant to at least one Group A drug.
- Patients who were physically and clinically stable enough to participate in interviews.

#### 2.3.2. Exclusion Criteria

- Patients with compromised clinical conditions that made participation in interviews challenging or unfeasible.
- Patients who had already completed XDR-TB treatment, as their recollection of counselling may be less accurate.

### 2.4. Sampling Technique

A purposive sampling technique was employed to ensure that participants met the study’s inclusion criteria, which included being adults diagnosed with XDR-TB and actively undergoing treatment at Brooklyn Chest Hospital. This approach enabled the selection of participants who could provide rich, relevant data about their experiences with information counselling and ototoxic symptoms. The sample size (N = 10) was determined based on the principle of thematic saturation, where no new themes emerged from the interviews, ensuring comprehensive coverage of the research objectives [33]. The selection process targeted XDR-TB patients meeting the inclusion criteria, with the assistance of nursing staff and healthcare workers at Brooklyn Chest Hospital who identified eligible participants and introduced the study. Healthcare providers at the hospital identified eligible participants based on the inclusion criteria, including age (≥18 years), clinical stability, and active treatment for XDR-TB. Once identified, eligible participants were approached by the researcher, who explained the study in detail, provided information sheets, and obtained written informed consent. Recruitment continued until data saturation was achieved, with no new themes emerging from participant interviews. A total of 10 participants were recruited, as this number allowed for data saturation in qualitative research, ensuring that new information from additional participants would no longer contribute significantly to the findings. The sample size for this study was determined using the principle of thematic saturation, which is commonly employed in qualitative research. Recruitment continued until no new themes emerged from participant interviews, ensuring that the data collected sufficiently captured the range of experiences and perspectives relevant to the study objectives. While the final sample size of 10 participants may appear small, it was adequate for achieving saturation given the depth of individual interviews and the focused scope of the study. In qualitative studies, smaller sample sizes are typical when the aim is to explore specific phenomena in depth rather than generalize findings to a broader population. The richness of the data collected from participants enabled the identification of key themes regarding information counselling and awareness of ototoxicity, addressing the research objectives comprehensively. This research site admits patients from diverse socio-demographic groupings (e.g., rural and urban areas).

### 2.5. Data Collection

#### 2.5.1. Instruments

Semi-structured in-depth interviews were conducted using an interview guide specifically designed for this study. Semi-structured in-depth interviews were employed to allow participants the flexibility to share their individual experiences while still covering specific themes relevant to the study’s objectives. The interview guide was carefully designed to address the following areas: (1) participants’ recollections and interpretations of any information counselling received about ototoxicity; (2) the timing of this counselling relative to treatment initiation; (3) the specific symptoms discussed during counselling sessions, as well as any subsequent guidance on symptom recognition and management; (4) personal experiences of ototoxic symptoms and their perceived impact on daily functioning and quality of life; and (5) understanding of and access to management and referral pathways if symptoms were experienced.

The interview guide was pretested in a pilot study with two participants, who did not form part of the main study, to refine question clarity and structure. Minimal changes were made following the pilot study. Only certain interview questions were modified to enhance participant comprehension, allowing for the provision of more accurate responses.

#### 2.5.2. Procedure

First, ethical clearance for the study was obtained from the Human Research Ethics Committee (HREC) of the University of the Witwatersrand (Clearance Certificate M240420) on 19th June 2024. Permission was also granted by Brooklyn Chest Hospital. All ethical considerations for research on human subjects, described in detail later, were adhered to. Second, interviews were conducted in private rooms within the hospital to ensure confidentiality and minimize interruptions. Each interview lasted approximately 20–30 min. The interviews were conducted in English or Afrikaans, depending on the participant’s language preference. Open-ended questions encouraged participants to share detailed personal insights, while closed-ended questions facilitated specific responses regarding whether they received information about ototoxicity and, if so, its timing and content. Lastly, with participant consent, interviews were audio-recorded to ensure accuracy in data capture and to allow for a detailed transcription process. Recordings were securely stored on a password-protected device accessible only to the research team [34]. The timing of interviews varied relative to participants’ treatment commencement. Some participants were interviewed during the early stages of their treatment, while others were at more advanced stages. This variation allowed for diverse insights but may have influenced participants’ ability to recall the timing and content of counselling and the emergence of ototoxic symptoms.

### 2.6. Data Analysis

An inductive thematic analysis was used to analyze the qualitative data, allowing for the identification and interpretation of recurring patterns and themes across participant responses. This approach is commonly employed in qualitative health research to generate insights that can inform patient-centered practices and improve healthcare delivery. Thematic analysis facilitated an understanding of how patients experienced information counselling, how they recognized and interpreted ototoxic symptoms, and how they navigated the healthcare system for symptom management. For thematic analysis, the approach outlined by Braun and Clarke [35] was employed. This six-phase method included: (1) familiarization with the data through repeated reading of transcripts, (2) generating initial codes by systematically identifying meaningful units within the text, (3) searching for themes by grouping related codes, (4) reviewing themes for coherence and relevance, (5) defining and naming themes to ensure clarity, and (6) producing the final report. NVivo software (10) was used to facilitate coding, organization, and retrieval of data during analysis, enhancing efficiency and transparency.

The thematic analysis resulted in the identification of key themes that aligned with the study objectives, providing insights into the timing, content, and effectiveness of information counselling for XDR-TB patients. Steps were taken to ensure rigor in analysis, including independent coding by two researchers and discussions to resolve discrepancies, ensuring a robust and credible interpretation of the data.

### 2.7. Ethical Considerations

This study was conducted with strict adherence to ethical standards to protect participants’ rights, maintain confidentiality, and uphold the integrity of the research process. Ethical considerations were structured around the principles of autonomy, beneficence, non-maleficence, and justice, which are foundational to ethical research in healthcare settings. Ethical approval was obtained from the University of the Witwatersrand Human Research Ethics Committee (Clearance Certificate M240420), with additional permission from Brooklyn Chest Hospital to conduct interviews on-site. Participants provided informed consent after receiving detailed information about the study’s purpose, their right to withdraw at any time, and the confidentiality of their responses. Consent forms were signed, and verbal consent was also confirmed for participants with limited literacy. Participation was entirely voluntary, and participants were assured that non-participation, or withdrawal would not affect their treatment.

To maintain confidentiality, pseudonyms were used in transcripts, and all data were securely stored on password-protected devices accessible only to the research team. Audio recordings and transcripts were kept separate from identifying information, with all data scheduled for secure disposal following institutional guidelines post-study. In cases where participants experienced emotional discomfort discussing treatment side effects, on-site counselling support was made available. This ethical approach, emphasizing respect, autonomy, and confidentiality, ensured that the study was conducted responsibly and aligned with the principles of beneficence and non-maleficence.

### 2.8. Rigor and Trustworthiness

To ensure the validity, reliability, and credibility of the findings, several strategies were employed to enhance both the rigor and trustworthiness of this study [36,37]. This approach included methodological and analytical rigor, as well as transparency in the research process, to achieve depth and consistency in understanding participants’ awareness and experiences regarding ototoxicity information counselling in XDR-TB treatment.

To ensure rigor and trustworthiness, including the pilot study, this study employed several strategies grounded in credibility, dependability, transferability, and confirmability. Data collection was systematic, with semi-structured interviews allowing for in-depth exploration of patient experiences. Data saturation was achieved when no new themes emerged, enhancing the credibility of the findings. Reflexivity was practiced through researcher journals to minimize bias, and peer review sessions validated the accuracy of coding and themes, ensuring a reliable and objective analysis.

For transferability, detailed descriptions of the study context and participant demographics were provided, allowing readers to assess applicability to similar settings. An audit trail documenting coding and theme development further reinforced transparency and confirmability. To enhance dependability, consistent procedures for data collection and thematic analysis were applied across all interviews, with external audits from a research supervisor validating the coherence and alignment of findings with the study’s objectives. These measures collectively strengthened the study’s credibility and ensured a robust and trustworthy analysis.

### 2.9. Data Management

Data management in this study was conducted with a focus on security, organization, and adherence to ethical standards [38]. All interviews were audio-recorded with participant consent and stored on a password-protected device accessible only to the research team. Interview recordings were transcribed verbatim and anonymized by replacing participant names with pseudonyms to maintain confidentiality. Transcripts and coded data were securely stored in encrypted digital files, with backup copies saved on institutional secure servers in compliance with university data protection policies. Physical documents, including consent forms, were stored in a locked cabinet separate from the electronic data. Only authorized members of the research team had access to the data throughout the study, ensuring secure and responsible handling of sensitive information. Upon completion of the research, all data will be retained for a specified period as per the university’s data retention guidelines and then securely disposed of, with digital files permanently deleted and physical documents shredded. This data management process ensures participant confidentiality and aligns with ethical standards for data integrity and security in qualitative research.

## 3. Results

### 3.1. Demographic Profile of the Sample

A total of 10 adults with XDR-TB participated in the study. The demographic characteristics of the participants, including gender and age, are summarized in Table 1.

The sample consisted of 60% female and 40% male participants, with the majority (60%) in the 20–29 age group. This distribution provides insight into the demographics of XDR-TB patients at the treatment facility in this study.

### 3.2. Receipt of Information Counselling on Ototoxic Effects

All participants were asked whether they had received any information counselling regarding the ototoxic side effects of their TB medications. The findings indicated that while 100% of participants reported receiving some form of information counselling, the depth and content of this counselling varied widely.

Only 30% of participants (*n* = 3) reported receiving comprehensive information counselling that specifically addressed ototoxic symptoms such as tinnitus, dizziness, and hearing loss. The remaining 70% of participants (*n* = 7) indicated that they received basic counselling, which included general information on side effects but lacked specific details about ototoxicity. This disparity in the depth of information counselling suggests that most participants may not have been adequately informed about the ototoxic risks associated with their TB medications, potentially impacting their ability to recognize and report early symptoms. These results suggest that while information counselling was provided to all participants, detailed information on ototoxic effects was limited.

### 3.3. Timing of Information Counselling

Participants were also asked about the timing of the information counselling they received relative to the start of their XDR-TB treatment. The timing was categorized into pre-treatment, during treatment, or both. The results are shown in Table 2.

As shown in Table 2, most participants (70%) received information counselling before starting their XDR-TB treatment, which allowed them to be somewhat prepared for potential side effects. However, 30% of participants only received counselling after treatment had already begun, possibly delaying their awareness and recognition of ototoxic symptoms. Only one participant (10%) received counselling both before and during treatment, which could reinforce awareness over time. This variability in counselling timing highlights potential inconsistencies in practice and suggests that many patients may not have been informed at an optimal time to promote early symptom recognition. These findings indicate that while most participants received counselling before treatment, a significant proportion did not receive information until after their treatment had begun, which may have delayed their awareness of ototoxic symptoms.

### 3.4. Content of Information Counselling

The study explored the specific content of the information counselling provided, focusing on three main themes: (1) general diagnosis and treatment information, (2) ototoxic side effects, and (3) management and follow-up instructions. Table 3 below provides a summary of responses for each theme.

Only 40% of participants recalled receiving information specifically about ototoxic side effects, and just 20% were informed of whom to consult if they experienced symptoms, highlighting a gap in the comprehensiveness of the counselling content.

### 3.5. Reported Occurrences of Ototoxic Effects

Participants were asked to report any ototoxic symptoms they had experienced since starting treatment. The results are presented in Table 4.

As shown in Table 4, half of the participants (50%) reported experiencing no ototoxic symptoms. Among those who did report symptoms, 20% experienced dizziness alone, while 30% reported both tinnitus and dizziness. The presence of these symptoms in 50% of participants highlights the potential impact of ototoxic TB medications as well as the need for heightened symptom awareness and monitoring, even though half of the sample did not report any symptoms. This variability in symptom reporting may reflect differences in individual susceptibility, awareness of symptoms, or variability in the information received about ototoxicity.

### 3.6. Timing of Management for Ototoxic Symptoms

The final objective examined the timing of audiological assessments, specifically baseline assessments and follow-up evaluations after treatment initiation. Table 5 summarizes the timing of these assessments. Only 40% of participants received a baseline audiological assessment before treatment began, while 50% had their baseline assessment conducted after treatment initiation. One participant (10%) did not receive any baseline assessment. Additionally, only 10% of participants received follow-up assessments after the baseline. The remaining 90% did not receive any follow-up testing, indicating a substantial gap in ongoing audiological monitoring for patients at risk of ototoxicity. This lack of follow-up assessments could hinder the early detection and management of ototoxic symptoms, suggesting an area where improvements in standard TB care could significantly benefit patient outcomes. These findings highlight gaps in early and consistent audiological monitoring, which may hinder the timely detection and management of ototoxic effects among XDR-TB patients.

### 3.7. Thematic Analysis Findings

The thematic analysis of participant responses identified several key themes related to the awareness and experiences of XDR-TB patients regarding the ototoxic effects of their TB medications. Each theme provides insight into specific aspects of the information counselling received by participants, its timing, content, and their experiences with ototoxic symptoms and management.

#### 3.7.1. Theme 1: Variability in the Depth of Information Counselling

Participants reported varying levels of detail in the information counselling they received regarding ototoxic effects. This theme highlights discrepancies in the depth of information, ranging from general discussions of side effects to specific details about potential auditory and vestibular symptoms. Comprehensive vs. Basic Counselling was found where while all participants reported receiving some form of information counselling, only 30% recalled receiving specific information on ototoxic symptoms such as tinnitus, hearing loss, and dizziness. The remaining 70% received more general information about side effects, lacking specific details on ototoxicity.

Participant A: “They told me the medication could affect my ears, but they didn’t go into specifics.”

Participant D: “They explained the side-effects of the medication that I’m going to take. Number 1, it can make your vision blurry and number 2, it damages your hearing.”

This theme suggests a need for a standardized approach to information counselling to ensure that all patients receive detailed, consistent information on ototoxicity.

#### 3.7.2. Theme 2: Inconsistent Timing of Information Counselling

The timing of information counselling was another significant theme, with participants indicating that the counselling was provided at various points before and during treatment, potentially impacting their preparedness for symptom monitoring. In total, 70% of participants received information counselling before treatment initiation, while 30% received counselling only after starting treatment. This delay in counselling may have reduced early symptom recognition and reporting among those informed later.

Participant F: “I got the information after I had started treatment, so I was already noticing some dizziness.”

Participant B: “They explained it to me before I started, which helped me watch for symptoms early.”

This theme indicates that pre-treatment counselling is beneficial for preparing patients to identify and report symptoms promptly, which could improve management and adherence.

#### 3.7.3. Theme 3: Limited Awareness of All Ototoxic Symptoms

Participants’ understanding of ototoxic symptoms was mostly limited to hearing loss, with less awareness of other symptoms like tinnitus and dizziness. This theme highlights a gap in the counselling content that impacts patients’ ability to recognize early signs of ototoxicity. Only 40% of participants were aware of specific ototoxic symptoms beyond hearing loss. Many were unaware of symptoms like tinnitus or vertigo, which often precede significant auditory damage.

Participant C: “They didn’t mention things like ringing in my ears; they only talked about hearing loss.”

Participant H: “They said that it will affect the ears and it might cause hearing loss and it can also affect the eyes, but not for everyone, but for most people.”

The limited scope of symptom awareness suggests a need for broader and more thorough counselling to enable early detection and reporting of all ototoxic symptoms.

#### 3.7.4. Theme 4: Lack of Clear Referral and Management Information

This theme emerged as participants expressed uncertainty about whom to approach if they experienced ototoxic symptoms. Several participants were unaware of the availability of audiological services or whom to consult when symptoms arose. One participant, for example, remarked, “I just thought I had to tell my doctor, but I wasn’t sure if they would do anything”. This lack of clarity reflects broader systemic challenges, including insufficient integration of audiological monitoring into TB care pathways. Integrating routine audiological referrals into TB treatment programs has the potential to significantly improve early detection and management of ototoxicity. These findings underscore the need for similar approaches in South Africa, including clear referral guidelines and expanded access to audiological services. Many participants were not informed about which healthcare providers were responsible for managing these side effects, indicating a gap in follow-up care guidance. Only 20% of participants reported being informed of whom to consult in the case of ototoxic symptoms, with others expressing confusion about their options, thus revealing uncertainty in management pathways.

Participant G: “They told me about the side effects, but I didn’t know I could go to an audiologist.”

Participant E: “I just thought I had to tell my doctor; I was just told to go the doctor should I experience anything.”

This lack of referral information could delay symptom management and contribute to patients’ uncertainty about seeking timely care for ototoxic symptoms.

#### 3.7.5. Theme 5: Experiences with Ototoxic Symptoms and Impact on Quality of Life

Participants reported a range of experiences with ototoxic symptoms, which impacted on their daily activities and quality of life. This theme highlights the practical implications of ototoxic effects on patients, emphasizing the need for effective counselling and management. 50% of participants experienced symptoms like dizziness or tinnitus, which affected their daily functioning and led to feelings of frustration and concern about their hearing and balance.

Participant I: “The dizziness makes it hard to even walk straight sometimes, but I thought it was part of the treatment.”

Participant J: “I keep hearing ringing sounds, which makes it hard to concentrate on things.”

The impact of these symptoms on patients’ lives illustrates the importance of early symptom recognition, comprehensive counselling, and accessible management options to mitigate these effects.

**Figure 1:**
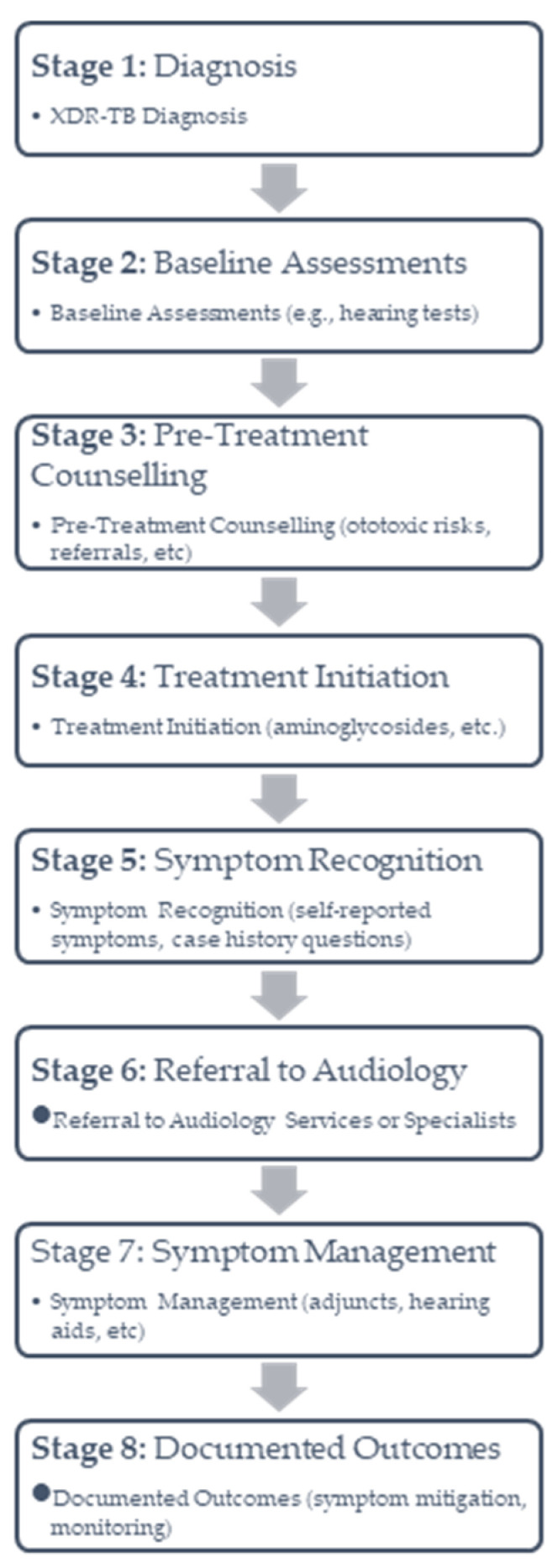
The pathway from XDR-TB diagnosis to the management of ototoxic symptoms.

Figure 1 below illustrates the pathway from XDR-TB diagnosis to the management of ototoxic symptoms. This flow highlights critical stages, including counselling, symptom recognition, and referral, as well as the gaps identified in this study, such as inconsistent counselling and limited access to audiological follow-up.

## 4. Discussion

This study aimed to explore the awareness of adults with XDR-TB regarding the ototoxic effects of their TB medications, specifically through the lens of information counselling, symptom recognition, and management within a South African context. The use of purposive sampling ensured that participants provided in-depth insights into their experiences with XDR-TB treatment, while reflexivity throughout the research process enhanced the credibility and rigor of the findings. By documenting assumptions and critically engaging with the data, the researchers minimized bias, allowing for a robust interpretation of the themes identified. The findings indicate critical gaps in information counselling, variability in the timing and depth of information provided, limited symptom awareness, and inconsistency in follow-up care. While this study highlights significant gaps in counselling practices, it does not explore the underlying causes of these gaps. It remains unclear whether these deficiencies stem from healthcare providers’ knowledge deficits or indifference to ototoxicity, workload constraints, or poor adherence to established counselling protocols. Identifying these root causes is essential for developing targeted interventions to improve the consistency and quality of patient counselling. Future research should investigate provider-level barriers, including training gaps and resource limitations, to address these systemic issues comprehensively.

While some participants reported receiving general information about TB treatment, specific counselling on ototoxicity risks was inconsistent. For example, one participant stated, “They told me the medication might have side effects, but they didn’t explain anything about my ears.” This lack of detail aligns with prior research, suggesting that counselling practices in South African public healthcare settings often prioritize adherence rather than comprehensive risk education. Comparatively, World Health Organization (WHO) standards emphasize the importance of pre-treatment counselling that includes detailed information on drug side effects, empowering patients to recognize and report symptoms early. The discrepancy between these standards and current practices suggests an urgent need for protocol standardization within South Africa’s healthcare system. These insights are particularly relevant in South Africa, where high TB and HIV burdens, combined with limited healthcare resources, exacerbate the complexities surrounding drug-resistant TB management. The discussion of the findings of this study is presented in accordance with the specific objectives of the study after the discussion of the demographic profile.

The demographic profile of the participants, primarily young adults (ages 20–29), reflects the impact of XDR-TB on a typically productive age group. This profile aligns with reports indicating that TB disproportionately affects young adults in South Africa, where socio-economic factors such as poverty, malnutrition, and overcrowded living conditions create conducive environments for TB transmission [2,39]. The predominance of females in the sample may also relate to gender-based health disparities, as women in certain socio-economic contexts are at heightened risk for TB due to factors such as caregiver roles and limited access to preventive healthcare [39]. Bearing in mind the qualitative nature of the study and thus the sample size precluding correlational analysis, the socio-demographic data in this study provide an important context for understanding the variability in participants’ awareness and experiences. Younger participants (ages 20–29) represented most of the sample (60%) and were more likely to report fewer or no ototoxic symptoms. This finding may be explained by their relatively shorter duration of exposure to ototoxic medications compared to older participants, who had been on treatment for longer periods. Additionally, gender dynamics may have influenced the reporting of symptoms, as women (60% of the sample) were more likely to discuss dizziness and balance issues, possibly reflecting differences in health-seeking behavior or symptom prioritization. These observations highlight the need for tailored information counselling that considers patients’ age, gender, and treatment stage to ensure comprehensive awareness and early symptom recognition. The observed demographic trends in symptom awareness highlight opportunities for tailoring interventions to specific patient groups. Younger participants (ages 20–29), who were less likely to report ototoxic symptoms, may benefit from targeted pre-treatment counselling that emphasizes early symptom detection, as they are at risk of delayed symptom recognition due to shorter treatment exposure. Similarly, female participants, who reported dizziness and balance issues more frequently, may require additional support in monitoring vestibular symptoms, as these can affect daily functioning and adherence to treatment. These findings suggest the need for age- and gender-specific counselling approaches, such as educational materials tailored for younger patients and vestibular rehabilitation strategies for individuals reporting dizziness. Furthermore, integrating these demographic insights into routine patient education sessions could enhance the relevance and effectiveness of counselling efforts, ensuring that patients across all demographic groups are equipped to manage the ototoxic risks associated with XDR-TB treatment.

The findings reveal significant variability in the information provided on ototoxicity, with only 30% of participants receiving detailed counselling on ototoxic symptoms. This limited counselling depth is consistent with previous research by Khoza-Shangase et al. [14], who noted that TB patients in South African public healthcare facilities often receive general information about side effects without specifics on ototoxicity. While the HPCSA guidelines emphasize the importance of pre-treatment information counselling for patients on ototoxic medications, there appears to be a gap between these recommendations and clinical practice. In South Africa’s resource-constrained healthcare system, providers often face high patient volumes, limiting their ability to deliver comprehensive, individualized counselling. Additionally, healthcare workers may lack sufficient training on ototoxicity, leading to general rather than specific information being shared. Studies suggest that comprehensive patient education enhances treatment adherence and symptom reporting, raising the need to improve the depth of information counselling for XDR-TB patients [13,14,19].

The study found that 70% of participants received information counselling before treatment, while others only received it during treatment. This delay in counselling timing potentially impacts patients’ ability to recognize and report ototoxic symptoms early. Receiving counselling prior to treatment is ideal, as it prepares patients for potential side effects and encourages early symptom identification, consistent with findings by Efendi et al. [40], who found that early counselling reduces psychological stress and promotes symptom monitoring. However, the inconsistent timing observed here is likely due to high patient caseloads and resource limitations within public healthcare facilities, where pre-treatment discussions are often abbreviated or delayed. Within the South African context, the timing of counselling is especially pertinent due to the high incidence of co-infections, such as HIV, which complicate TB treatment and further strain healthcare resources. Delays in counselling may inadvertently lead to delayed symptom recognition, impacting timely interventions and potentially worsening the degree of ototoxic damage.

Participants’ understanding of ototoxic symptoms was primarily limited to hearing loss, with limited awareness of other symptoms like tinnitus and dizziness. This finding is concerning, as tinnitus and dizziness often precede significant hearing loss in patients on ototoxic drugs. Previous studies have highlighted similar gaps in awareness, finding that TB patients frequently underreport early signs of ototoxicity due to limited understanding of these symptoms [2,4,5,14,18]. The limited awareness of diverse ototoxic symptoms in this study reflects broader issues within the South African healthcare system, where audiology services are often inaccessible to many patients, especially in rural and under-resourced areas [21]. Audiologists, who play a crucial role in patient education on ototoxic risks, are scarce in public hospitals, which may contribute to the inadequate awareness of all potential symptoms. Additionally, language and literacy barriers prevalent in South Africa further complicate patient understanding, necessitating the development of culturally and linguistically tailored educational materials [41].

The study highlighted a notable lack of clear guidance on whom to consult if ototoxic symptoms arise, with only 20% of participants informed about appropriate referral pathways. This lack of structured referral information suggests that patients may not fully understand how or when to seek care for ototoxic symptoms, a finding that aligns with the observations of Govender et al. [12], who noted inadequate coordination among healthcare providers regarding ototoxicity management in South Africa. Inadequate referral information is particularly concerning given the need for early audiological intervention to mitigate ototoxic damage. Within South Africa’s public healthcare system, many patients rely on general practitioners for primary care and may not have direct access to audiology services [42]. This issue highlights the need for multidisciplinary collaboration in TB management, ensuring that patients are not only informed of ototoxic risks but also aware of the available healthcare resources and specialists, such as audiologists, who can support symptom management.

Half of the participants reported experiencing ototoxic symptoms such as dizziness and tinnitus, which significantly impacted their daily activities and quality of life. These findings align with previous studies which emphasize the psychosocial and functional burden of ototoxicity, particularly in the South African context where access to rehabilitative services is limited [4,5,14,15]. Symptoms like tinnitus and dizziness can disrupt balance, concentration, and social interactions, affecting patients’ adherence to treatment and their overall mental health. For patients dealing with both XDR-TB and the side effects of ototoxic medications, the lack of adequate support services exacerbates their distress. South African public hospitals often have limited audiology facilities, and rehabilitative services are primarily concentrated in urban areas, making it difficult for patients in rural regions to receive timely and appropriate care. Given the high burden of TB and HIV, combined with the scarcity of specialized services, ototoxicity management should be prioritized as an integral component of TB care in South Africa to reduce the long-term impact on patients’ lives.

The WHO has called for comprehensive management of drug-resistant TB, including patient education on treatment side effects [43]. However, implementation within the South African context remains challenging. Effective ototoxicity management for XDR-TB patients in South Africa will require strategic initiatives, such as integrating audiology services into TB treatment facilities, training healthcare providers on ototoxicity, and creating standardized counselling protocols. Given the limited audiology infrastructure identified in this study, several actionable recommendations can address these gaps:

- Leverage mHealth for Counselling and Follow-Up: mHealth technologies can be used to deliver educational messages about ototoxic symptoms and provide reminders for follow-up appointments, particularly in rural areas.
- Establish Community-Based Audiological Screening Programs: Training community health workers to conduct basic hearing assessments and refer patients with symptoms to audiologists can expand access in under-resourced regions.
- Standardize Pre-Treatment Counselling Protocols: Developing national guidelines that require detailed pre-treatment counselling, including visual aids and culturally tailored materials, can improve patient understanding of ototoxic risks.

These strategies could facilitate symptom screening and early intervention, ensuring patients receive timely referrals to specialists even in resource-limited settings. Implementing standardized information counselling practices, as well as utilizing accessible, culturally appropriate materials, could also enhance patient understanding of ototoxic symptoms and encourage proactive engagement with their care.

While aminoglycosides such as amikacin and kanamycin remain integral to many XDR-TB regimens in resource-constrained settings, newer medications like bedaquiline and delamanid have emerged as alternatives with reduced ototoxic risk profiles [1,4,6]. Bedaquiline, for example, has demonstrated significant efficacy in reducing treatment duration and adverse effects. Unlike aminoglycosides, bedaquiline has no documented ototoxicity, making it a promising option for minimizing auditory and vestibular complications in XDR-TB patients [6]. However, its use in South Africa is often limited by cost and availability, particularly in public healthcare facilities. Adjunct therapies such as otoprotective agents have also been explored, though evidence supporting their routine implementation remains inconclusive [18]. Future integration of these alternatives into treatment protocols could reduce ototoxic risks while maintaining therapeutic efficacy.

Aminoglycosides are known for their concentration-dependent bactericidal activity, but their pharmacokinetics pose challenges due to accumulation in cochlear and vestibular hair cells, resulting in irreversible damage. Current HPCSA guidelines recommend baseline audiological assessments and regular monitoring during aminoglycoside use to mitigate these risks [11]. However, findings from this study indicate a lack of alignment between policy and practice, with participants reporting inconsistent counselling and follow-up monitoring. This gap highlights the need for enhanced implementation of existing policies, particularly in resource-constrained healthcare settings like South Africa.

Pharmacovigilance, the proactive monitoring and management of adverse drug reactions, offers a critical opportunity to balance the efficacy of XDR-TB treatment with patient safety. Integrating pharmacovigilance into routine TB care could involve establishing centralized reporting systems for ototoxicity, training healthcare providers to identify and address early symptoms, and incorporating newer, less toxic drugs into standard regimens as they become more accessible. This approach aligns with WHO recommendations for strengthening TB care through patient-centered models and improved safety monitoring. By prioritizing pharmacovigilance, South Africa could enhance the quality of care for XDR-TB patients while reducing the burden of treatment-related adverse effects.

In South Africa, ototoxicity monitoring and the management of other treatment-related adverse events, such as anemia or peripheral neuropathy, are guided by hospital-specific resources and practices. At Brooklyn Chest Hospital, audiological monitoring is included as part of the XDR-TB treatment protocol, with baseline audiological assessments recommended before the initiation of ototoxic medications. However, the findings from this study indicate that such assessments are inconsistently implemented, often delayed, or not followed by routine monitoring. Resources for managing adverse events like ototoxicity include access to audiologists and ENT specialists, although these services are often limited to larger urban hospitals. For non-audiological adverse events such as anemia or peripheral neuropathy, patients are referred to relevant specialists or provided with adjunct treatments, such as vitamin supplementation or iron therapy, depending on availability.

Despite these provisions, systemic resource constraints, high patient volumes, and limited staffing hinder the consistent application of these protocols. These challenges highlight the need for comprehensive resource audits and staff training programs to ensure alignment between hospital policies and clinical practice.

This study has several limitations that should be considered when interpreting the findings. First, the sample size was relatively small (N = 10) and limited to one specialized TB treatment facility, which may affect the generalizability of the results. While the qualitative approach provided in-depth insights, a larger sample across multiple facilities would enhance the representativeness of findings within the broader population of XDR-TB patients in South Africa. Second, the study relied on self-reported data from participants, which may be subject to recall bias, particularly concerning the details and timing of information counselling received. Some participants may have had difficulty recalling specific aspects of counselling or may not have fully understood certain medical information, which could impact the accuracy of responses. Additionally, the study’s reliance on self-reported symptoms to explore patient experiences of ototoxicity may not fully capture the true prevalence of hearing loss due to the absence of audiometric data, and the reliance on non-specific symptoms like dizziness introduces the risk of misclassification. Future studies should incorporate objective assessments, such as pure-tone audiometry and otoacoustic emissions, to provide a more accurate evaluation of ototoxic effects in XDR-TB patients. Another limitation is the single point of data collection in a cross-sectional design, which captures only a snapshot of participants’ experiences and awareness at one stage of their treatment journey. Longitudinal data collected at multiple points throughout treatment could provide a more comprehensive view of how awareness and symptom management evolve over time. Additionally, the recruitment process relied on nursing staff and healthcare workers to identify eligible participants, which may have introduced selection bias. Healthcare providers might have unintentionally excluded patients with poor rapport, those who experienced severe adverse events without prior counselling, or those with poor adherence to treatment protocols. Additionally, the study did not record the total number of eligible patients screened or excluded, limiting transparency regarding participant selection. Lastly, although interviews were conducted in participants’ preferred language (English or Afrikaans), language diversity within the South African context is bigger that these two languages. With the languages spoken by the majority of Black South Africans being excluded in the study, some linguistic or cultural nuances may not have been fully captured, especially if participants were not fluent in the interview language and/or did not speak English or Afrikaans. Future studies incorporating additional languages and diverse cultural contexts could further enhance understanding and applicability of findings in diverse South African healthcare settings.

## 5. Conclusions

This study explored the awareness of ototoxic side effects among adults with XDR-TB undergoing treatment in South Africa, with a focus on the role of information counselling in preparing patients to recognize and manage these effects. The findings highlight substantial gaps in the depth and timing of information counselling, with many patients receiving only general information on side effects and limited guidance on specific ototoxic symptoms like tinnitus and dizziness. The variability in the timing of counselling, coupled with a lack of structured referrals for symptom management, emphasize the need for standardized practices that ensure patients are adequately informed and supported throughout their treatment journey. In the South African context, where TB and HIV rates are high, and public healthcare resources are limited, ototoxicity management poses unique challenges. Limited access to audiology services, especially in rural and under-resourced areas, further complicates the early detection and intervention necessary to mitigate long-term hearing loss and balance issues in patients treated with ototoxic medications. Given the significant functional and psychosocial impact of ototoxic symptoms, there is a critical need for targeted interventions that enhance patient awareness, improve symptom reporting, and provide timely management support.

For XDR-TB patients facing the dual burden of a severe illness and potential hearing damage from ototoxic medications, early and thorough information counselling is essential. Not only does it support symptom awareness and early reporting, but it also facilitates adherence to complex TB regimens. The South African healthcare context, characterized by resource constraints and high patient volumes, presents unique challenges to delivering consistent, high-quality patient education. However, addressing these gaps in information provision and audiological follow-up is critical to preventing and managing ototoxic effects, improving patient outcomes, and enhancing quality of life. Based on the study findings, a few recommendations can be proposed.

Firstly, information counselling protocols must be standardized. The South African audiology profession must develop and implement standardized, detailed information counselling protocols across TB treatment centers, particularly for drug-resistant cases. Counselling should specifically cover the ototoxic side effects of TB medications, including detailed information on symptoms such as tinnitus, hearing loss, and dizziness, as well as the importance of reporting early signs. These protocols must ensure that counselling is provided pre-treatment and reinforced during treatment, allowing patients time to internalize the information and actively monitor their symptoms. Secondly, audiological monitoring and referral pathways must be enhanced. This means that audiology services must be integrated more effectively into the TB treatment framework by ensuring baseline assessments are conducted before treatment initiation and that regular follow-up evaluations are available to monitor for ototoxic effects. Furthermore, clear referral pathways must be established to guide patients experiencing ototoxic symptoms towards audiology specialists or other appropriate healthcare providers, especially in public healthcare facilities where resources may be stretched. Thirdly, training and resources for healthcare providers must be increased. Training for healthcare providers on ototoxicity in TB treatment, including identifying symptoms, delivering comprehensive counselling, and establishing referral networks with audiologists must be provided. This training should be aligned with HPCSA guidelines and made available to all staff involved in TB care. Additionally, healthcare providers must be equipped with culturally and linguistically appropriate materials to support effective patient communication across South Africa’s diverse population. Fourthly, community-based and mobile health interventions must be implemented. Community health workers and mHealth interventions must be leveraged to improve outreach and monitoring in under-resourced or rural areas. Community health workers trained in identifying ototoxic symptoms can support early reporting and assist in directing patients to appropriate services. The audiology profession can also consider tele-audiology as a feasible approach for monitoring and managing ototoxicity remotely, reducing barriers to access in regions with limited audiology services. Lastly, research and policy development must be prioritized. Further research into the prevalence and impact of ototoxicity among TB patients in South Africa must be encouraged to better understand the scope of this issue and inform evidence-based policy. Collaborative efforts between policymakers, researchers, and healthcare providers can support the implementation of the already existing national HPCSA guidelines for ototoxicity management. Future research should also explore the effectiveness of telehealth approaches, mHealth applications, and other scalable interventions that can support the needs of XDR-TB patients in resource-limited settings. Research on patient outcomes following the implementation of standardized information counselling could provide valuable data to inform policy changes and best practices. Policies should prioritize ototoxicity monitoring as a standard element of TB treatment, with funding allocations to support the required infrastructure and staff training across all provinces.

Improving ototoxicity awareness, prevention, and management among XDR-TB patients can have far-reaching implications for patient outcomes, quality of life, and healthcare system efficacy in South Africa. By addressing the current gaps in counselling, monitoring, and referral systems, the healthcare system can provide more patient-centered and effective support for individuals navigating the complexities of TB treatment. Standardizing these efforts can enhance patient autonomy, reduce the risk of undiagnosed hearing loss, and ultimately contribute to a more resilient healthcare system prepared to meet the challenges of drug-resistant TB.

The recommendations proposed in this study, such as leveraging mHealth technologies, integrating community-based audiological screening, and standardizing counselling protocols, are scalable across other resource-limited settings with similar challenges. In high TB-burden countries such as Ethiopia, India, and Nigeria, where healthcare resources are constrained, mHealth interventions could provide cost-effective solutions for improving patient education and follow-up care. Training community health workers to perform basic hearing assessments and serve as patient advocates can extend access to audiological services in rural and underserved regions. Standardized protocols, incorporating culturally and linguistically tailored counselling materials, can ensure consistency and relevance in patient education, even in diverse healthcare systems. By adapting these strategies to local contexts, resource-limited settings worldwide can address gaps in ototoxicity awareness, monitoring, and management, ultimately improving patient outcomes in XDR-TB care.

## Figures and Tables

**Table 1:** Demographic characteristics of participants (N = 10).

Characteristic	Category	Number of Participants	Percentage (%)
Gender	Male	4	40%
	Female	6	60%
Age Group	20–29 years	6	60%
	30–39 years	2	20%
	40–49 years	2	20%

**Table 2:** Timing of information counselling (N = 10).

Timing of Counselling	Number of Participants	Percentage (%)
Before Start of Treatment	6	60%
During Treatment	3	30%
Both Before and During Treatment	1	10%

**Table 3:** Summary of counselling content on ototoxic effects (N = 10).

Content Category	Information Received (%)	Sample Responses
Diagnosis and Treatment	100%	“I was told about my diagnosis and the importance of treatment adherence.”
Ototoxic Side Effects	40%	“They mentioned I might experience hearing problems.”
Management and Follow-Up	20%	“I was told to report any dizziness or hearing issues to the doctor.”

**Table 4:** Reported ototoxic symptoms among participants (N = 10).

Reported Symptoms	Number of Participants	Percentage (%)
Dizziness	2	20%
Tinnitus and Dizziness	3	30%
No Symptoms	5	50%

**Table 5:** Timing of audiological assessments among participants (N = 10).

Timing of Assessment	Number of Participants	Percentage (%)
Baseline Pre-Treatment	4	40%
Baseline Post-Treatment Start	5	50%
No Baseline Assessment	1	10%
Follow-Up Assessments	1	10%

## Data Availability

Data supporting the findings of this study are available within the paper; the rest of the data that support the findings of this study are not openly available due to reasons of sensitivity, and are available from the corresponding author upon reasonable request.
